# Preoperative Glucagon‐Like Peptide‐1 Receptor Agonist Treatment to Allow Safe Laparoscopic Left Pancreatectomy in Extreme Obesity: The First Report

**DOI:** 10.1155/cris/9201182

**Published:** 2026-01-07

**Authors:** Giulia Canali, Gregoire Herfeld, Gerlinde Averous, Philippe Baltzinger, Pietro Addeo

**Affiliations:** ^1^ Hepato-Pancreato-Biliary Surgery and Liver Transplantation, Pole des Pathologies Hépatiques et Digestives, Hôpital de Hautepierre-Hôpitaux Universitaires de Strasbourg, Université de Strasbourg, Strasbourg, France, unistra.fr; ^2^ Department of Endocrinology, University of Strasbourg, Hôpital de Hautepierre-Hôpitaux Universitaires de Strasbourg, Université de Strasbourg, Strasbourg, France, unistra.fr; ^3^ Department of Pathology, University of Strasbourg, Hôpital de Hautepierre-Hôpitaux Universitaires de Strasbourg, Université de Strasbourg, Strasbourg, France, unistra.fr

**Keywords:** GLP1 analogs, laparoscopy, obesity, pancreatic resections, weight loss

## Abstract

Obesity is a challenging condition for pancreatic surgery, and some authors recommend delaying pancreatic resection for non‐malignant pancreatic tumors in obese patients. We present a case of a 45‐year‐old woman with a body mass index (BMI) of 56 who was surgically treated in our department for a mucinous cystadenoma discovered during preoperative work‐up for bariatric surgery. To decrease the risk involved in pancreatic surgery, a glucagon‐like peptide‐1 receptor agonist was administered for 6 months, which led to a weight loss of 20 kg and a BMI of 48 at the time of surgery. A laparoscopic left splenopancreatectomy was performed within 7 months of the diagnosis. The postoperative length of stay was 19 days. Pathology confirmed that the tumor was mucinous cystadenoma with mild dysplasia. As of 17 months later, the patient is doing well and has lost an additional 10 kg.

## 1. Introduction

The worldwide obesity epidemic is rapidly growing and is leading to a greater prevalence of surgical candidates who are obese. Obese individuals have been shown to have a lower life expectancy and greater risk of death from all causes compared to the general population [1], and obesity is universally recognized as a challenging condition for various types of surgery, particularly abdominal surgery. This also applies to pancreatic surgery, and several studies have reported higher rates of morbidity among patients with obesity undergoing this type of surgery [2, 3]. This is related to the greater susceptibility to developing postoperative complications and the increased complexity of surgery in such patients, especially if minimally invasive approaches are used [4].

In this context, it has been described as increased rates of surgical‐site infection, pancreatic fistula, delayed gastric emptying, intraoperative bleeding, and postoperative respiratory failure, resulting in longer lengths of hospital stays and higher costs [5–9]. For these reasons, some authors recommend delaying pancreatic resection for non‐malignant tumors [10, 11]. We present a case of a 45‐year‐old woman with a history of extreme obesity who was treated with a glucagon‐like peptide‐1 receptor agonist (GLP‐R) before surgical treatment for a pancreatic tumor.

## 2. Case Presentation

The patient was a 45‐year‐old woman who had a past medical history of extreme obesity complicated by hypertension, type 2 diabetes, and dyslipidemia. The patient was referred to our unit for the surgical treatment of a pancreatic tumor, which was discovered during preoperative work‐up for bariatric surgery. She had previously undergone a sleeve gastrectomy, but after consistent weight loss, she regained 30 kg and had reached a body mass index (BMI) of 56 (weight 132 kg, height 152 cm). Magnetic resonance imaging showed a large 5‐cm cystic tumor on the pancreatic tail located close to the splenic hilum (Figure 1). Endoscopic ultrasonography confirmed the presence of a 5‐cm mass, which was unilocular and suggestive of mucinous cystadenoma with a single mural nodule. Given the size and the presence of a mural nodule, surgery was indicated [12].

Figure 1Preoperative magnetic resonance imaging showing a large pancreatic cystic tumor in an obese patient (blue arrow) (A); the patient had central obesity (B).

**Figure:**
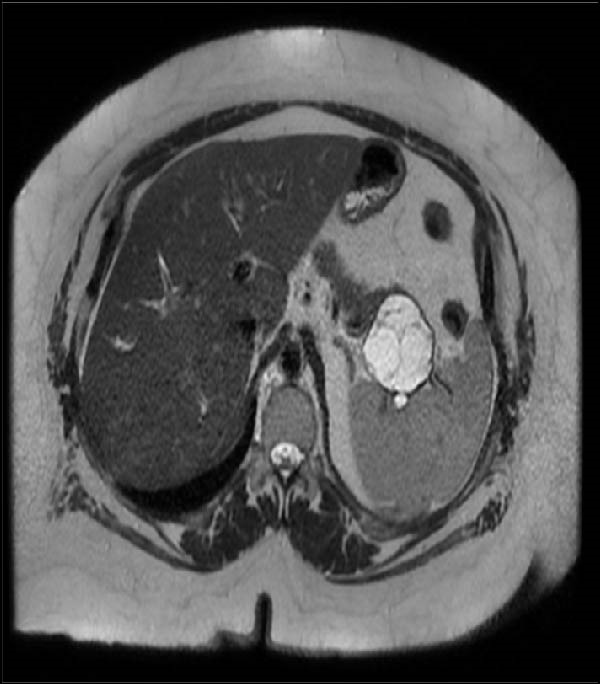
(A)

**Figure:**
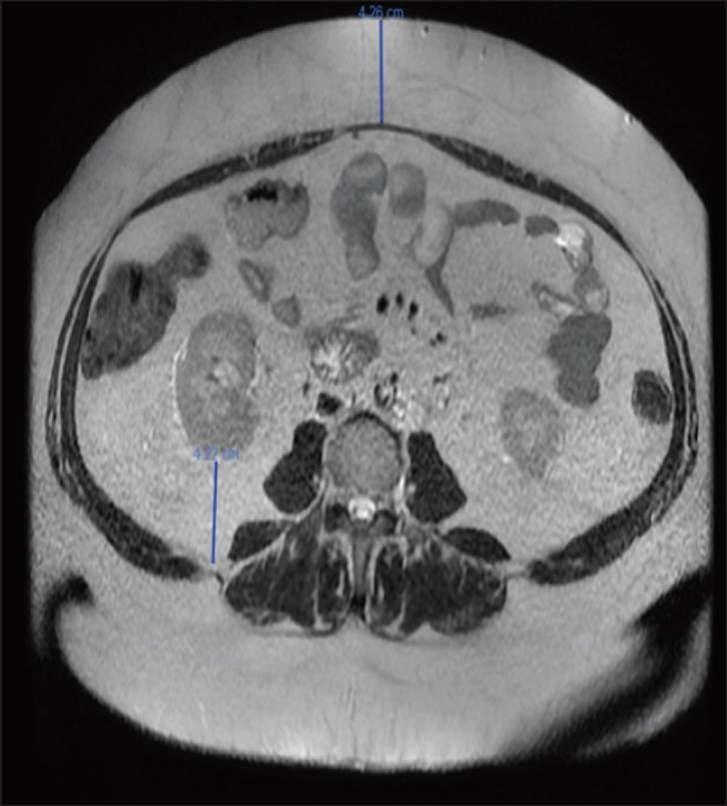
(B)

Considering the risks of pancreatic resection in an obese patient and the probable benign nature of the mass, we decided to postpone surgery until the patient reached an ideal weight. The case was discussed with the endocrinology team to find a way to achieve sufficient weight loss in the shortest time possible. The patient was then put on a weight loss program that included dietary restrictions and increased physical activity, as well as pharmacological treatment with a GLP‐R. She started Semaglutide at a dose of 0.25 mg once weekly for the first 4 weeks, with subsequent dose increases every 4 weeks to 0.5 mg, 1 mg, 1.7 mg, and finally 2.4 mg. The overall tolerance of the drug was excellent, with the exception of some mild nausea during the first week following the initial 0.25 mg dose, but no further gastrointestinal symptoms occurred in the following weeks. Over 6 months, the patient lost 20 kg and achieved a BMI of 48, which allowed us to perform surgery within 7 months after the diagnosis with fewer surgical risks for the patient. As advised by the anesthesiologist, she did not receive her injection the week before surgery. No insulin bridging was required during this period, and her diabetes was well controlled preoperatively. A laparoscopic approach was employed to perform a laparoscopic left splenopancreatectomy. There were no notable problems during surgery. The postoperative course was complicated by a grade‐B pancreatic fistula, which did not require any specific treatment besides leaving the drain in place up to day 15. Drain amylase values at POD3, 6, 9, 12, and 15 were 2896, 1804, 2886, and 4919 with a drain output of 50, 60, 30, and 10 mL per day. The postoperative length of stay was 19 days. Semaglutide was resumed at a dose of 1.0 mg starting 1 month after surgery. The postoperative HbA1c was 5.8%. Pathology confirmed that the mass was mucinous cystadenoma with mild dysplasia, but there was no adenocarcinoma (Figure 2). As of 17 months later, the patient is doing well and has lost an additional 10 kg.

Figure 2Hematoxylin and eosin stain (A) showing mucinous cystadenoma with ovarian‐type stroma (B). Immunohistochemistry stain showing cell positivity for smooth muscle actin (SMA) (C), desmin (DES) (D), estrogen (ER) (E), and progesterone (PR) (F).

**Figure:**
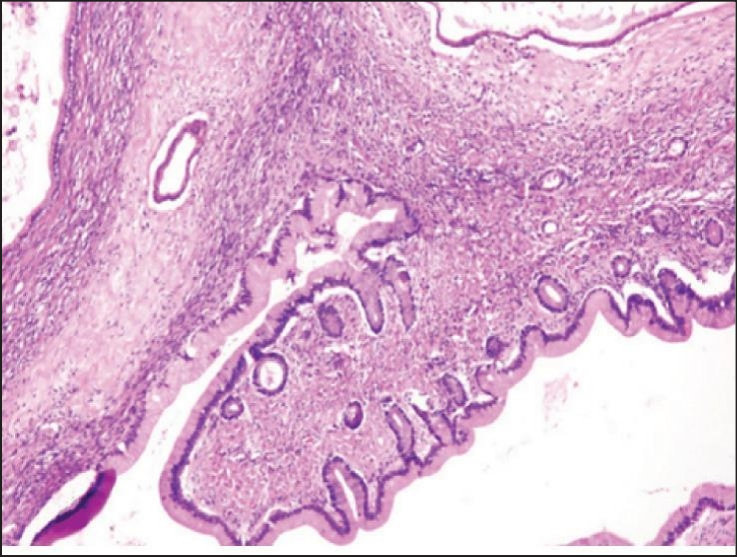
(A)

**Figure:**
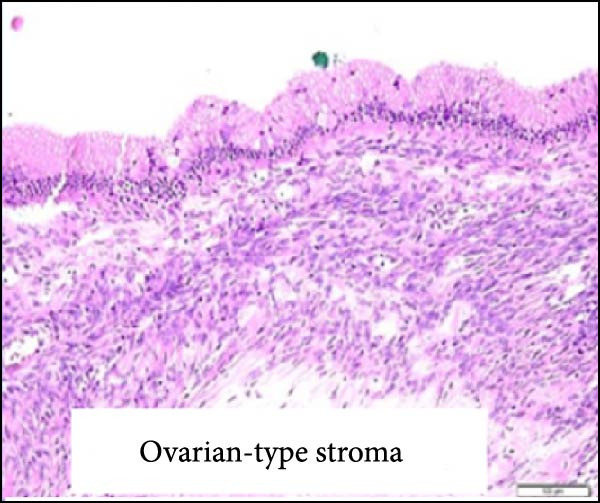
(B)

**Figure:**
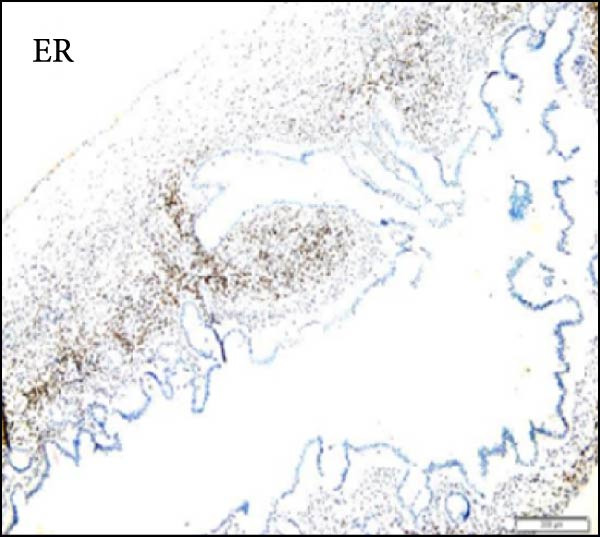
(C)

**Figure:**
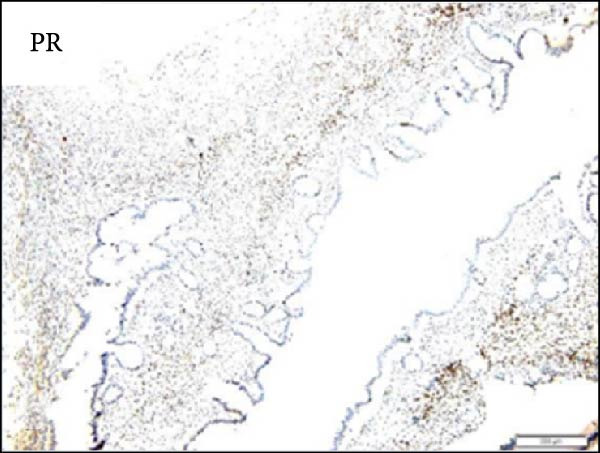
(D)

**Figure:**
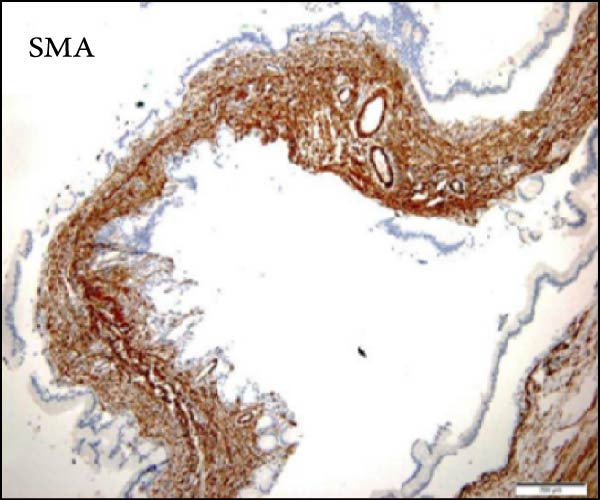
(E)

**Figure:**
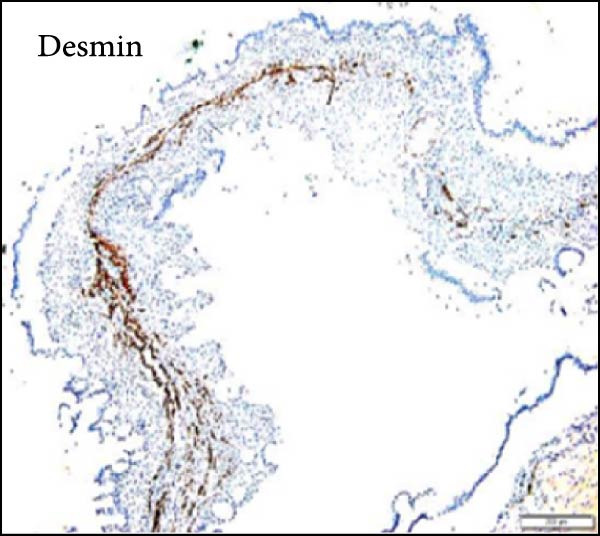
(F)

## 3. Discussion

Obesity is spreading worldwide and is making surgery more and more challenging. Multiple studies concerning pancreatic surgery have described increased technical difficulties and increased morbidity in obese patients. The origin of these issues is multifactorial. First, the simultaneous deposition of fat into the subcutaneous area, the omentum, and the perirenal areas increases the anteroposterior diameter of the abdominal cavity, which increases the difficulty of reaching and exposing the pancreatic region [11].

Second, pancreatic fat deposition has been recognized as a risk factor for pancreatic fistula, leading to more clinically significant pancreatic leaks. Third, patients with obesity could develop more clinically significant non‐surgical morbidity due to obesity‐related comorbidities such as diabetes and cardiovascular disease. They also have more risk of developing postoperative respiratory failure, wound infection, and prolonged hospital stay [3, 13]. All of these factors result in greater rates of intraoperative bleeding, postoperative medical and surgical complications, and readmissions.

There are several options that can be adopted to reduce the risks related to obesity in patients in need of pancreatectomy. One option is delaying surgery until after the completion of a weight loss program [10]. This option can be safely adopted by patients who need pancreatic resection for non‐malignant pathology, but the patient must be compliant, and variable amounts of time before surgery are needed. A second option is bariatric surgery [13]. However, this option is characterized by additional surgical risk. A third option is a combination of a weight loss program and a GLP‐R, which does not involve any additional surgical risk and allows substantial weight loss that can reduce surgical morbidity.

The use of a GLP‐R has been associated with improved control of diabetes and substantial weight loss [14]. This drug has both a central and peripheral activity. Indeed, it activates GLP‐1 receptors in the central nervous system, stimulating satiety neurons and diminishing the rewarding sensation typically associated with dietary consumption. This results in reduced food intake and increased energy expenditure. Peripherally, it slows gastric emptying, enhancing feelings of fullness after meals; it promotes the release of gut hormones like ghrelin, peptide YY, and cholecystokinin that further suppress appetite; it improves insulin sensitivity and reduces triglycerides and LDL‐cholesterol levels, leading to better mobilization and utilization of fat stores for energy and consequent reduction in ectopic fat storage [15].

Because of its efficacy for weight loss, its use is rapidly growing, even among non‐diabetic patients. Recently published evidence shows that it has benefits when used as a prehabilitation tool for weight loss in obese patients undergoing elective hernia repair [16].

This report has shown the first clinical use of a preoperative GLP‐R in a patient with obesity and a non‐malignant pancreatic tumor to induce weight loss and allow a safe pancreatic resection. In cases of pancreatic surgery, a GLP‐R enables effective weight loss and reduces the time to surgery compared with lifestyle modifications alone. Nevertheless, it does not increase the risk or discomfort for the patient and gives surgeons another possible strategy to optimize patient treatment. It is, however, important to mention the risk of delayed gastric emptying and the consequent potential risk of aspiration during anesthesia associated with this drug, which should prompt physicians to take appropriate precautions when managing patients receiving this treatment [17].

## 4. Conclusions

Preoperative weight loss is fundamental in pancreatic surgery to reduce intraoperative and postoperative complications and to reduce readmission rates. This can be achieved by using different approaches. This clinical report has suggested a novel approach to achieve substantial weight loss using GLP‐R agonists for obese surgical candidates. This strategy is both effective and safe. However, further studies are needed to evaluate the cost‐effectiveness of such medications in such prehabilitation settings and their application in other types of abdominal surgeries.

## Ethics Statement

In our study, an approval from the Institutional Review Board was deemed unnecessary for the following reasons: This report presents a single case with no interventions or deviations from standard care protocols, posing minimal risk to the patient’s welfare. The patient voluntarily consented to the publication of this case report after being informed of its purpose. Throughout the process, principles of respect for autonomy, beneficence, and non‐maleficence were upheld. Confidentiality and anonymity have been maintained in adherence to ethical standards.

## Disclosure

All authors read and approved the final manuscript.

## Conflicts of Interest

The authors declare no conflicts of interest.

## Author Contributions

**Giulia Canali, Gregoire Herfeld, Gerlinde Averous, Philippe Baltzinger,** and **Pietro Addeo**: conceptualization, formal analysis, methodology, project administration, visualization, validation, supervision,writing – original draft, and writing – review & editing.

## Funding

The authors received no specific funding for this work.

## Data Availability

The data that support the findings of this study are available on request from the corresponding author. The data are not publicly available due to privacy or ethical restrictions.

## References

[1] Adams K. F. , Schatzkin A. , and Harris T. B. , et al.Overweight, Obesity, and Mortality in a Large Prospective Cohort of Persons 50 to 71 Years Old, New England Journal of Medicine. (2006) 355, no. 8, 763–778, 10.1056/NEJMoa055643, 2-s2.0-33747870163. 16926275

[2] Del Chiaro M. , Rangelova E. , Ansorge C. , Blomberg J. , and Segersvard R. , Impact of Body Mass Index for Patients Undergoing Pancreaticoduodenectomy, World Journal of Gastrointestinal Pathophysiology. (2013) 4, no. 2, 37–42, 10.4291/wjgp.v4.i2.37. 23755369 PMC3676538

[3] Lovasik B. P. , Kron P. , Clavien P. A. , Petrowsky H. , and Kooby D. A. , Pancreatectomy and Body Mass Index: An International Evaluation of Cumulative Postoperative Complications Using the Comprehensive Complications Index, HPB. (2019) 21, no. 12, 1761–1772, 10.1016/j.hpb.2019.04.006, 2-s2.0-85066240491. 31153835

[4] Balduzzi A. , van der Heijde N. , and Alseidi A. , et al.Risk Factors and Outcomes of Conversion in Minimally Invasive Distal Pancreatectomy: A Systematic Review, Langenbeck’s Archives of Surgery. (2021) 406, no. 3, 597–605, 10.1007/s00423-020-02043-2. PMC8106568 33301071

[5] Tsai S. , Choti M. A. , and Assumpcao L. , et al.Impact of Obesity on Perioperative Outcomes and Survival following Pancreaticoduodenectomy for Pancreatic Cancer: A Large Single-Institution Study, Journal of Gastrointestinal Surgery. (2010) 14, no. 7, 1143–1150, 10.1007/s11605-010-1201-3, 2-s2.0-77953690563. 20431978

[6] Dindo D. , Muller M. K. , Weber M. , and Clavien P.-A. , Obesity in General Elective Surgery, The Lancet. (2003) 361, no. 9374, 2032–2035, 10.1016/S0140-6736(03)13640-9, 2-s2.0-0037840171. 12814714

[7] Robinson J. R. , Marincola P. , Shelton J. , Merchant N. B. , Idrees K. , and Parikh A. A. , Peri-Operative Risk Factors for Delayed Gastric Emptying After a Pancreaticoduodenectomy, HPB. (2015) 17, no. 6, 495–501, 10.1111/hpb.12385, 2-s2.0-84929274464. 25728447 PMC4430779

[8] Benns M. , Woodall C. , Scoggins C. , McMasters K. , and Martin R. , The Impact of Obesity on Outcomes Following Pancreatectomy for Malignancy, Annals of Surgical Oncology. (2009) 16, no. 9, 2565–2569, 10.1245/s10434-009-0573-7, 2-s2.0-68949218320. 19557479

[9] Williams T. K. , Rosato E. L. , and Kennedy E. P. , et al.Impact of Obesity on Perioperative Morbidity and Mortality After Pancreaticoduodenectomy, Journal of the American College of Surgeons. (2009) 208, no. 2, 210–217, 10.1016/j.jamcollsurg.2008.10.019, 2-s2.0-58249101412. 19228532

[10] Di Gioia A. , Giuliani T. , and Marchegiani G. , et al.Pancreatoduodenectomy in Obese Patients: Surgery for Nonmalignant Tumors Might be Deferred, HPB. (2022) 24, no. 6, 885–892, 10.1016/j.hpb.2021.10.018. 34801400

[11] Vanbrugghe C. , Ronot M. , and Cauchy F. , et al.Visceral Obesity and Open Passive Drainage Increase the Risk of Pancreatic Fistula Following Distal Pancreatectomy, Journal of Gastrointestinal Surgery. (2019) 23, no. 7, 1414–1424, 10.1007/s11605-018-3878-7, 2-s2.0-85052143780. 30120668

[12] van Huijgevoort N. C. M. , del Chiaro M. , Wolfgang C. L. , van Hooft J. E. , and Besselink M. G. , Diagnosis and Management of Pancreatic Cystic Neoplasms: Current Evidence and Guidelines, Nature Reviews Gastroenterology & Hepatology. (2019) 16, no. 11, 676–689, 10.1038/s41575-019-0195-x, 2-s2.0-85073836181. 31527862

[13] Umemura A. , Sasaki A. , and Nitta H. , et al.A Novel Second-Stage Surgical Strategy for Severely Obese Patient with Pancreatic Neuroendocrine Tumor: A Case Report, Surgical Case Reports. (2022) 8, no. 1, 10.1186/s40792-022-01484-9, 125. 35754064 PMC9234015

[14] Morissette A. and Mulvihill E. E. , Obesity Management for the Treatment of Type 2 Diabetes: Emerging Evidence and Therapeutic Approaches, Journal of Pharmacy & Pharmaceutical Sciences. (2024) 27, 10.3389/jpps.2024.13065, 13065. 38903652 PMC11186996

[15] Moiz A. , Filion K. B. , Tsoukas M. A. , Yu O. H. Y. , Peters T. M. , and Eisenberg M. J. , Mechanisms of GLP-1 Receptor Agonist-Induced Weight Loss: A Review of Central and Peripheral Pathways in Appetite and Energy Regulation, The American Journal of Medicine. (2025) 138, no. 6, 934–940, 10.1016/j.amjmed.2025.01.021. 39892489

[16] Spurzem G. J. , Broderick R. C. , and Ruiz-Cota P. , et al.GLP-1 Receptor Agonists Are a Transformative Prehabilitation Tool for Weight Loss in Obese Patients Undergoing Elective Hernia Repair, Surgical Endoscopy and Other Interventional Techniques. (2025) 39, no. 1, 440–447, 10.1007/s00464-024-11308-6. 39369100 PMC11666797

[17] Ushakumari D. S. and Sladen R. N. , ASA Consensus-Based Guidance on Preoperative Management of Patients on Glucagon-Like Peptide-1 Receptor Agonists, Anesthesiology. (2024) 140, no. 2, 346–348, 10.1097/ALN.0000000000004776. 37982170

